# A qualitative study of the interaction experiences between family caregivers and community nurses for disabled elderly people at home

**DOI:** 10.1186/s12877-023-03917-y

**Published:** 2023-04-21

**Authors:** Panpan Guo, Shanfeng Zhang, Meilan Niu, Panpan Wang, Ling Li, Chuqiao Wu, Di Zhao, Rui Ma, Peng Wang

**Affiliations:** 1 Scool of Nursing and Health, Zhengzhou University, Zhengzhou, China; 2 Experimental Center for Basic Medicine, Zhengzhou University, Zhengzhou, China; 3 Department of Pharmacology, Medical School of Huanghe Science and Technology University, Zhengzhou, China; 4 The Ninth People’s Hospital of Zhengzhou, Zhengzhou, China; 5 Zhengzhou, China

**Keywords:** Disabled elderly people, Family caregiver, Community nurse, Interaction, Qualitative

## Abstract

**Background:**

Family members are currently the main caregivers of the disabled elderly people at home. With declining health and increasing frailty, caregiving of disabled elderly people becomes a task of family caregivers in conjunction with community nurses. Interaction between family caregivers and community nurses can effectively improve the quality of home care for the disabled elderly people. This study aimed to investigate the interaction experiences between family caregivers and community nurses for disabled elderly people at home.

**Methods:**

This research was a study of qualitative descriptions based on semi-structured face-to-face interviews. This study was to purposefully select family caregivers of the disabled elderly and community nurses in Zhengzhou city, Henan Province and explore the interaction patterns between them. Directed content analysis method was used to generate qualitative codes and identify themes.

**Results:**

A total of 12 interviews were completed, including 7 family caregivers and 5 community nurses. Four themes were identified: 1) Information interaction; 2) Emotional interaction; 3) Practical interaction; 4) Factors that promote and hinder the interaction.

**Conclusions:**

It was found that the interaction between family caregivers and community nurses was not optimistic. Lack of communication and collaboration between community nurses and caregivers. Providing a new perspective that we can develop and implement intervention to facilitate positive interactions, which will reduce the burden of family caregivers, bring the highest quality of care to older adults with disabilities and improve the quality of care for disabled elderly people.

**Trial registration:**

Registered in the Chinese Clinical Trial Registry on April 19, 2021, number ChiCTR2100045584.

## Background

Population aging has become a worldwide phenomenon, and the concern of elderly care is expanding worldwide [1]. The seventh census of population in China shows that there are 190 million people aged 65 or above, accounting for 13.5% of the total population [2]. Due to the decrease of mortality rate and the extension of life expectancy, the number of the elderly is increasing at the rate of 5.96 million per year. It is predicted that by 2050, China's population above 60 years of age may be reach 498 million [3]. With advancing age, it means the continuous degradation of various physiological functions. This population trend also means that the proportion of people with mental or physical disabilities who are unable to carry out activities of daily living increases [4]. According to statistics, by 2019, there will be more than 40 million disabled and semi disabled elderly people in China, which is expected to reach more than 60 million by 2030 and 96 million by 2050 [5].

“Disability” is an integrative concept that represents the state of incomplete self-care due to various reasons such as old age, disease or physical and mental disorders [6, 7]. With the aging of population, the care problems caused by the increase of the disabled elderly have become increasingly prominent. Most of the disabled elderly are more vulnerable to the pressure brought by the new environment due to the influence of traditional ideas and physical weakness and aging [8, 9], and are unwilling to choose an unfamiliar environment outside the family and community [10, 11].

To meet the needs of the disabled elderly, the government advocates and encourages home-based care [12]. Both family caregivers and community nurses play an important role in the care of the disabled elderly at home. Their mutual cooperation can effectively improve the quality of home care for the disabled elderly [13]. In the context of home-based care, engagement of community can not only meet the demands of the disabled elderly who are eager for stay-at-home, but also help families provide life care and ensure the quality of care for the disabled elderly [14]. Compared with institutional elder care, community participation in home-based care has the advantages of low cost and high efficiency, which make the demand for community care services of the disabled elderly continue to increase [15]. At present, the family members, with children and spouses as the main body, are still the main providers of home care for the disabled elderly [16]. With the increase of age and disability, the care needs of the disabled elderly at home also increase [17]. However, the trend of family’s centralization and miniaturization leads to the decrease of available care manpower in the family [18]. In addition, the burden of long-term care and the lack of time and energy further weaken the function of current care manpower; furthermore, family caregivers are generally deficient in professional caring knowledge and tools, which can not satisfy the requirements of technical care services, resulting in the deficiency of care workforce and ability, which directly affects the quality of care for the disabled elderly [19, 20]. Therefore, in the process of home care for the disabled elderly, the support provided by the family is very limited, and it is difficult to fully realize the long-term care for the disabled elderly. The family's demand for the assistance of long-term professional care from the community is also growing [21].

The collaboration between family caregivers and community professionals' can effectively improve the mental health level of the disabled elderly [22]. Other studies have shown that the establishment of professional community medical and nursing service institutions can take over the care of the disabled elderly when their families can not take care of them, so as to avoid their worries [23, 24]. On the other hand, professional community service institutions can ensure the disabled elderly to seek medical treatment at the first time in case of physical discomfort or emergency, and reduce the treatment risk of the disabled elderly [25]. Community medical staff provide professional and refined services. Family caregivers can replace professionals, provide less technical informal care services. Family caregivers and community nurse coordinate and cooperate, which enable the disabled elderly to live in their familiar environment and receive professional care services [26]. The interactive relationship between community nurses and caregivers provides caregivers with a good way to vent their bad emotions and gain emotional support. Studies have shown that community nurses have established a stable trust relationship with them during continuous family visits, and that caregivers are willing to open up to their difficulties and dissatisfaction, and proactively express their various emotional and social needs [27].

Henan province is located in central China, and Zhengzhou city is the capital of Henan Province, with a large population base and a severe aging trend. In recent years, the Zhengzhou Municipal government has gradually attached great importance to the construction of the elderly care service system. By 2021, Zhengzhou has built 67 street elderly care service centers and 657 community day care centers [28]. With the continuous promotion of national basic public health services, the number of community nurses in China is increasing. At the end of 2020, there were 219,574 community nurses in China [29]. Community nurses need to provide basic care and other health care services to individuals, families, communities and social groups, including disease and injury prevention, health promotion, disability rehabilitation [30]. There is no unified standard for charging fees for home care services [31]. Community nurses often work independently for in home hospital beds, home care and other services [32]. However, Community nurses focus on providing services to the elderly and lack awareness of attention and communication with caregivers. Therefore, there few opportunities for communication between caregivers and community nurses [33, 34]. Therefore, it is necessary to value the interaction relationship between community nurses and family caregivers.

The effective interaction between family caregivers and community nurses is very important in the process of caring for the disabled elderly at home. However, the previous studies on the interaction relationship between family caregivers and medical staff mostly focused on aging service agencies and hospitals, and did not pay much attention to the contexts of family caregiving in community settings [35–37]. Therefore, this study aims to explore the interaction experience of them in caring for the disabled elderly at home from the perspective of family caregivers and community nurses, so as to provide reference significance for future related research.

## Methods

### Study design and setting

This qualitative research was conducted between March and June 2022. Qualitative data were collected from family caregivers and community nurses of homebound disabled elderly people in Zhongyuan District, Zhengzhou City, Henan Province. All participants provided written informed consent. The study has been approved by the ethical review committee of Zhengzhou University (ZZUIRB2021-15).

We used semi-structured [38] face to face interviews, basic qualitative descriptions and a content analysis to understand the characteristics of interaction community nurses and family caregivers of disabled elderly in China. Qualitative descriptions aim to provide a comprehensive summary of events in terms of the participants’ experiences and perceptions [39]. A directed content analysis is used to examine face-to-face interaction. We believe that a directed content analysis is an ideal method. A directed content analysis requires the theoretical framework or theory to be identified before the data analysis begins [40]. We chose Jun-E Liu’s Nurse-patient communication model to guide analysis of the interaction between community nurses and family caregivers. Jun-E Liu’s Nurse-patient communication model includes the following 3 key structures: emotion, information, and behavior [41]. We report our study design and findings according to the Consolidated Criteria for Reporting Qualitative Research (COREQ) checklist [42].

### Researcher characteristics

The two investigators (PG and DZ) are female nursing students with a health care background, trained in conducting and analysing qualitative interviews. No relationship existed with the participants prior to the interviews. One investigator conducted the interviews, the other investigator was responsible for recording and time management and both participated in coding the transcripts. The two investigators had masters level expertise in qualitative data collection and analysis.

### Participants

The family caregiver should be: 1) at least ≥ 18 years old; 2) be the family members of the disabled elderly people (aged ≥ 60 years and Katz Index score < 6) and taking care of the elderly as the main caregiver for more than 3 months. The community nurses should be: 1) at least 18 years old; 2) the service and management personnel of relevant institutions in the community who often have direct contact with the elderly and provide door-to-door services; 3) having been engaged in community service for more than one year. Exclusion criteria included cognitive disorders, unwillingness to participate or to complete the interview, and temporary employees.

### Data collection

Two primary investigators conducted semi-structured face to face interviews with community nurses and family caregivers of disabled elderly between March and June 2022. Interviews were conducted at Community Health Services Center and family caregivers’ homes. Participants were recruited through purposive samplings from the Linshanzhai Community Health Services Center in Zhengzhou City, Henan Province. Firstly we contacted the community nurses and interviewed them after consent. After the interview, community nurses led us to find family caregivers of disabled elderly people who met the inclusion criteria within their jurisdiction. Explain the purpose and significance of the study, and ask if they were willing to participate in the interview. Degree of disability of the elderly was measured by Katz Index Scale [43, 44]. A score 6 represents complete independence, 4 ~ 5 represents mild disability, 2 ~ 3 represents moderate disability, and 0 ~ 1 represents severe disability. Prior to the interview, participants were informed the research content and purpose, as well as confidentiality protections. They signed an informed consent form if they agreed to participate and granted us permission for recording. No non-participants were present during interviews; no repeat interviews were carried out. The content of interviews included participants’ demographic characteristics (i.e., gender, age, highest education level, and living status) and interaction experience, through the following questions (See Table 1 for interview guidelines). Interviews were recorded and transcribed verbatim and transcripts were identified and verified for accuracy against audio recordings. The sample selection was continued until no other themes or sub-themes appeared.

**Table 1:** Interview guidelines

Participants	Questions
Family Caregivers	①Has the community nurses ever assisted you? What is it? ②What assistance would you like from community nurses? ③How do you view your relationship with community nurses? ④How do you feel about your interaction with community nurses? What aspects are involved? ⑤Have you ever had a conflict with a community nurses? How did this happen?
Community Nurses	①Have you ever provided assistance to family caregivers of disabled older people? What is it? ②How do you feel about your relationship with your family caregiver? ③How do you feel about your interaction with family caregivers of disabled older people? ④ What do you think are involved in the interaction with family caregivers? ⑤Have you had any conflicts with family caregivers? How did this happen?

### Analyses

Two researchers agreed to end the participant recruitment process when no novel information seemed to emerge from participant interviews. Interview records were stored on a secured device to fully protect the privacy of participants. The audio-taped interviews were listened repeatedly and transcribed verbatim. Before the formal analysis, the interviewees were coded with letters and numbers F1-F7, N1-N5 to process personal sensitive information such as their names. Then directed content analysis method was used to extract the effective content, code, classify and simplify the relevant content, extract the theme and return to the interviewees for confirmation, and finally refine the interview theme. The data analysis focuses on interaction experience of the disabled elderly family caregivers and community nurses. The coding scheme was based on the three key elements of Jun-E Liu’s Nurse-patient communication model: emotion, information, and practice [41]. These elements provided provided an analytical perspective of the interactions between community nurses and family caregivers of disabled elderly.

An analysis team comprised two analysts (PG and DZ). Firstly, the data were open coded using a line-by-line coding process. The two analysts engaged in data immersion to be fully familiar with the data. A letter, word, sentence, or a part of the page was coded as the unit of analysis. The analysts carefully read the transcripts to identify evidence of the theoretical derived codes. Repetitive words, concepts and phrases were recognized. Then, the codes were grouped into broader categories reflecting the characteristics of interactions between community nurses and family caregivers. Finally, the codes and categories of each transcript was compared between the analysts, and differences were discussed and resolved to ensure consistency of the research team [45]. The analysis continued until no new information emerged from the interviews (data saturation) [46].

To ensure credibility, the two coders consulted with each other to address ambiguities or disagreement on methodological issues or data analysis. The findings were reviewed by a third, senior researcher with expertise in this field of study, to confirm whether the descriptions accurately reflected the interaction experience of the disabled elderly family caregivers and community nurses.

## Results

### Demographic characteristics

In this qualitative study, the interviews were conducted between March and June 2022. Following the principle of data saturation, data collection was ceased once saturation was achieved. Finally a total of 12 interviews were completed, including 7 family caregivers and 5 community nurses. The demographic information for the each participants is illustrated in Tables 2 and 3.

**Table 2:** Demographic information of family caregivers (*N* = 7)

number	gender	age	Relationship with the disabled elderly	Time of caring (year)	Degree of disability of the elderly
F1	male	66	son	4	moderate
F2	female	73	daughter	6	moderate
F3	male	82	spouse	5	severe
F4	female	63	daughter	3	severe
F5	male	79	spouse	3	moderate
F6	female	59	daughter	5	severe
F7	male	50	son	3	mild

**Table 3:** Demographic information of community nurses (*N* = 5)

number	Gender	age	Education level	Professional title	Years worked in community
N1	female	59	Associate degree	Senior nurse	30
N2	male	34	Bachelor's degree	Junior nurse	10
N3	female	28	Bachelor's degree	nurse	5
N4	female	33	Bachelor's degree	nurse	9
N5	female	36	Associate degree	Junior nurse	15

**Fig. 1:**
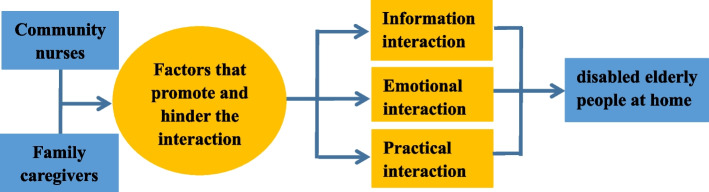
The interaction between community nurses and family caregivers of disabled elderly

After the interview, the recording data of seven disabled elderly family caregivers and five community nurses were transcribed in time. The interviews ranged from 30 to 90 min. The theme framework was proposed using the interaction model (Fig. 1). Based on the data collection and directed content analysis, four overarching themes were identified (See Table 4 for major themes and categories):

**Table 4:** Themes and categories

Themes	Categories
Information interaction	a. Mutual communication being the premise of establishing a good interactive relationship
	b. Information sharing being the basis of information interaction
Emotional interaction	a. Perceive positive emotional interaction
	b. Perceive negative emotional interaction
Practical interaction	a. Providing assistance in medical treatment
	b. Health management of disabled elderly people
Factors that promote and hinder the interaction	a. Factors that promote the interaction
	b. Factors that hinder the interaction

### Theme 1: Information interaction

#### Mutual communication being the premise of establishing a good interactive relationship

Communication is a mediator of the relationship between transmitter and receiver and a vehicle at the root of human behaviours. More specifically, in all verbal and non-verbal communication, human beings transmit information, ascribe meaning to the information, and adjust their behaviours according to that meaning and to the context in which they find themselves. Persons who communicate with one another influence each other mutually in a process of continuous feedback. When communication leads to harmonious cooperation between two interlocutors, this is referred to as a positive circular pattern of communication (CPC). It is useful for observing interactions and interdependence among family members and between nurses and family members [47]. The duration of services provided by community nurses each time was very limited, and in-depth communication was not possible. Although they wanted to get information from medical staff, they gave up communication for fear of disturbing and bothering community nurses.

F4: "They (community nurses) seem to have a lot of things to do. They just leave after asking a few questions. They don't have time to sit down and chat with me, but I really need information and help. I really hope someone can spend more time explaining it to me."

F1: "we also know that people (community nurses) are very busy. Generally, I don't want to give people trouble. Sometimes when I can't help it, I take the initiative to call them."

At present, the communication form between caregivers and community nurses is mainly telephone help, and caregivers hope to have a variety of communication forms with community nurses to achieve effective communication. Most family caregivers said they hoped to increase the frequency of communication with community nurses.

F7: "Occasionally they calls to inquire about the old man's health. If the community can visit us more, or hold more lectures in the community, this form of face-to-face communication is better."

F2: "I don't have much contact with community nurses, and they seldom come here. If only they could come here often."

#### Information sharing being the basis of information interaction

The family caregivers and community nurses both are experts in their own fields.with a mutual and equal relationship. The family caregivers are experts on their own life and with respect to their experiential knowledge of the illness. The community nurses, on the other hand, are experts at understanding the illness experience because of their education and practice experience. Good interaction involves comprehensive attention to the disabled elderly, which also includes the attention paid to family caregivers in terms of disseminating information and answering questions. Information and experience sharing contributes to the care of the disabled elderly people. Most of the family members of the disabled elderly are not medical personnel, and they want to get more information about home care. The content and form of information provided need to meet the individual needs of caregivers. Caregivers are not only eager to acquire care knowledge, but also hope to provide an information exchange platform for health consultation.

F1: "I take care of the elderly at home. I need to know more about what I can and can't do."

F4: "I think there should be some written or video materials instead of simple oral information, and the professional terms should be avoided, which should be easy to understand, so that we older people can understand."

F6: "I think we can set up a wechat group. We can consult in it if we have problems, which is equivalent to resource sharing. I think it's very good."

### Theme 2: Emotional interaction

#### Perceive positive emotional interaction

The good interaction between the community nurses and family caregivers brings a positive experience to the caregivers. Caregivers expect to communicate with community nurses as much as possible about the disease. When the caregivers' efforts are affirmed and praised by the community nurses, the caregivers can get self satisfaction and sense of honor, which will help to provide better care for the disabled elderly people.

F5: "There is a community nurse who is very good. She often asks her about some diseases by phone. She has a good attitude and is very grateful to her."

F2: "It's good to interact with the community nurses, and their encouragement enhances my confidence in care."

F3: "A while ago, when the community nurse came to change the gastric tube, she also praised me for taking good care of my wife. I was very happy and proud."

#### Perceive negative emotional interaction

However, many caregivers said that at present, the focus of community nurses is the disabled elderly, and there is a lack of attention and care for caregivers. Because caregivers have been under high psychological pressure for a long time, they have heavy psychological load and can't be released. Therefore, caregivers urgently need some psychological counseling and psychological counseling to relieve caregivers' pressure and maintain their good emotional state.

F1: "Everyone thinks this is what I should do. It's normal to take care of my father. Community nurses has paid no attention to me. Because I'm not a patient."

F5: "Sometimes when I talk to community nurses, they have an arrogant and *cold attitude. It makes me sad."*

F3: "I don't get enough rest because of taking care of my wife, which makes me *very upset. The community nurse would always point out the inadequacies of my work, which made me very stressed and miserable."*

### Theme 3: Practical interaction

#### Providing assistance in medical treatment

The health status of disabled elderly people determines that they should often go to relevant medical institutions. However, due to the lack of opportunities to communicate with the outside world, the family caregivers of the disabled elderly are socially isolated. Secondly, It is difficult for family caregivers to take the elderly with activity difficulties to see a doctor alone. For example, they faced physical functional limitations and difficulties caused by their built environment. Therefore, how to see a doctor is a difficult problem for caregivers, which requires community nurses to coordinate hospital visits.

F3: "When my father feels sick. It's not convenient to take him out. We live in apartment buildings without elevators. I always contact the community nurse first for help, since we are so close."

N2: "If the patient needs treatment, we can bring the doctor to the door to give him a preliminary diagnosis to see if he needs to be hospitalized. This saves him tossing back and forth."

F2: "Now the days of stay in major hospitals is very short. If we want to continue hospitalization for observation and conditioning, we must transfer to community hospitals, but the transfer is very troublesome. I really hope community nurses can help us."

#### Health management of disabled elderly people

Disabled elderly people often suffer from long-term chronic diseases, so they need long-term condition monitoring, such as measuring blood pressure, blood glucose, vital signs, etc., but some family caregivers are not competent for this task. Most of the family members have no care experience, and feel at a loss in the face of patients' diseases and disease-related complications. They hope to obtain nursing guidance and assistance related to rehabilitation exercise for the disabled elderly people. However, the assistance and resources available from community nurses were limited and could not meet family caregivers’ needs. There is no good collaboration developed between community nurses and family caregivers.

F5: " Sometimes the community nurses come to the home to check the blood pressure, blood sugar and medication status of elderly people for free. That's what I really need. Then I hope they can help us see if the medicine is right."

F1: "I hope the community nurse can guide the elderly rehabilitation exercise, or show me how to do it. I want to learn, but I don't know who to learn from. Let the child find information on the Internet to learn so, and I don't know whether it's professional or not."

F3:"The nurses in the community are very good and responsible. The stomach tubes inserted by my wife are all made by the community nurses. It's inconvenient for me to go upstairs and downstairs. They let me sit and wait and help me go through the formalities. Thanks to them."

### Theme 4: Factors that promote and hinder the interaction

#### Factors that promote the interaction

Community nurses are expected to be active and assertive in supporting family members in illness situations. Mutual collaboration is of importance. A positive attitude toward collaboration of community nurses-family caregivers can enhances the interaction. The family caregivers reported appreciation of community nurses’ interpersonal skills even more than their professional skills.

N1: "I often interact with family caregivers through phone calls or home visits. I think this is an interesting job, which can build a good relationship with family caregivers and facilitate my future work."

Some caregivers said that the participation of community nurses in the care of disabled elderly at home was low. The participation of community nurses should be increased, which will provide more opportunities for interaction.

F2: "It seems that taking care of the elderly is my task alone. Community nurses rarely participate in it, and the service content they provide is particularly little. They come occasionally, that is, taking blood pressure or something, and notifying the elderly to have a physical examination every year."

F5: "Only when we take the initiative to seek help will they come over, and the service provided is not particularly timely. They seldom take the initiative to come over, and I feel unfamiliar with them."

Trust is the cornerstone of a good relationship, and the family caregiver's trust in the professional competence of the community nurse is conducive to their interaction.

F3: "They (community nurses) are professional. The community nurses know a lot and I'm sure they can handle all the usual nursing problems."

In addition, community nurses and family caregivers should be tolerant of each other, reduce conflicts, take the health of the disabled elderly as the common goal, and actively promote cooperative relations.

F7: "However, we understand and respect each other, because our ultimate goal is for the health of the elderly."

N2: “Due to the differences in nursing knowledge, sometimes I have difficulties in communicating with family caregivers. I understand them very well and try to explain to them patiently to avoid conflicts.”

#### Factors that hinder the interaction

Community nurses-family caregivers interaction is complicated by busy work schedules, lack of time, and negative attitudes. Community nurses are busy with their daily work and do not have enough time and energy to contact and communicate with family caregivers of disabled elderly. Community nurses always overemphasize on efficiency manifests itself in haste, excessive concentration on procedures and ignoring the family’s wishes, which impedes the creation of a good interactive relationship between community nurses and family caregivers.

N2: "We do a lot of work, including physical examinations of the elderly and follow-up for diabetes and hypertension. Usually, many family caregivers take the initiative to contact me for door-to-door service. I'm too busy to interact with my family caregivers. There's no way."

The charging standard is a factor affecting interaction. Some caregivers worry that the charges are too high. They choose to take care of the disabled elderly themselves, rarely contact and interact with the community nurses, and refuse the help and services provided by the community nurses.

N1: "An elderly man was bedridden and needed to be fed through a gastric tube.His daughter bought a stomach tube online to save money and inserted it herself. But she was not professional like us. But she's not as professional as we are.At that time, she inserted the trachea, causing the old man to infect his lungs."

In addition, community nurses have deficiencies in the health management of the disabled elderly and did not receive interaction training. The professional quality of community nurses was not balanced. There is no professional community nurse dedicated to caring the disabled elderly and interacting with family caregivers.

N3: "We don't have community nurses dedicated to the health management of the disabled elderly. If there are, it will be better. Community nurses also have a weak grasp of the professional knowledge of the disabled elderly. The quality of personnel should be improved and training should be strengthened.

## Discussion

With the deepening of aging population degree, long-term care for disabled elderly has become a challenging problem gradually. On the one hand, the disabled elderly hope to stay at home [11]; Nevertheless, due to the weakening of family function and the limited support family providing [10], it is difficult to fully realize the home-based long-term care for the disabled elderly, which eventually leads to the increasing demand for community care [21].

Family caregivers and community-based care service can combine the advantages of both good family care and community professional services [26]. Some studies have shown that creating a positive and friendly social environment can promote the positive interaction between family and community, effectively improve the quality of care for the disabled elderly at home, and reduce the burden of family and society [48–50].

Studies have reported the importance of the quality of social interactions between family and formal care providers in residential care settings, and less is known about such relationships in community-based care settings in which the majority of disabled older adults [51, 52]. Semi-structured was conducted interviews with community nurses and family caregivers of disabled elderly to collect information regarding their experiences and attitudes toward interaction. Based on the data collection and directed content analysis, four overarching themes were identified: Information interaction, Emotional interaction, Practical interaction, and Factors that promote and hinder the interaction.

Collaboration is one of characteristics of social interaction between family caregivers and community-based service providers [35, 53]. If family caregivers are able to build a collaborative and reciprocal relationship with the community caregivers, which will enable them to better provide care for the disabled elderly people, while reduce the burden on the family caregivers [54, 55]. And being well connected can enhance this collaborative relationship between family caregivers and community nurses [48, 56]. However, at present, caregivers have access to limited the help and resources of community nurses, and no building good collaboration relationship. Other family caregivers said community nurses often had very limited time of services provided to unable to communicate deeply with them. Therefore, caregivers hope to increase the frequency of communication with community nurses, as well as provide multiple forms of communication to achieve effective communication [57, 58].

Getting professional support includes emotional support, information support, and practical support. Family caregivers who perceived higher levels of support reported significantly higher levels of satisfaction with professional providers [35]. Family caregivers, who are mostly non-medical practitioners, want the community to provide an information-sharing platform for health counseling to get more information about the care of the disabled elderly. In terms of practical help, there are still deficiencies in the quality and quantity of services provided by community nurses. As many disabled elderly people suffer from long-term chronic diseases, community nurses should visit regularly to monitor their health status for a long time [59]. Due to the health problems of the disabled elderly, the lack of relevant care skills and heavy burden of caregivers, they are eager to have professional nursing staff to provide services to reduce their burden [60]. Moreover, caregivers also raised the problem of difficulty in seeing a doctor, hoping that community nurses can assist in seeing a doctor. However, community-based service agencies often focus on physical aspects of caregiving, such as the number of hours served and tasks completed, rather than the social or emotional aspects [61]. Caregivers have heavy psychological load and cannot be released under high psychological pressure for a long time [62, 63]. Therefore, caregivers urgently need some psychological counseling and psychological counseling to relieve caregivers' pressure and maintain their good emotional state. Previous studies showed the importance of provider support on the psychological well-being of family caregivers [64]. When the caregivers' efforts are affirmed and praised by community nurses, their self satisfaction and sense of honor will be improved, which will help to provide better care for the disabled elderly.

Factors that promote and hinder the interaction the interaction. At present, the participation of community nurses in the care of disabled elderly at home is low. In the interactions between nurses and family caregivers, nurses usually lack initiative [65]. In order to promote the interaction between caregivers and community nurses, the participation of community nurses should be increased, and community nurses should be more active in the home care of the disabled elderly. Moreover, community nurses and family caregivers should tolerate each other, reduce conflict, take the health of the elderly as a common goal, and actively promote partnerships [66]. However, there are insufficient community nurses in the health management of the disabled elderly, and the professional quality of community nurses is uneven. There are also some family caregivers with poor physical condition or low cooperation and low attention, resulting in inadequate care. These all will hinder interaction between family caregiver and community nurses. Interventions to facilitate positive interactions such as support and collaboration between family and nursing home care providers have been developed and tested [67]. Such interventions can be adapted for community-based care settings. Family members are more likely to provide a larger share of caregiving tasks in community settings compared to nursing home settings [35, 68].

In light of the implications of the findings of this study, future research could further advance this field by developing interventions to enhance interactions between home caregivers and community nurses to help them to effectively collaboration and support each other. Besides, government and social organizations should create and provide a suitable environment where family caregivers and community nurses can focus on strengthening their interactive relationships to bring the highest quality of care to older adults with disabilities.

## Strengths and limitations

This was the qualitative study to explore the interaction experiences between family caregivers and community nurses for disabled elderly people at home. We collected the voices and feelings about interaction directly from participants. Despite this study’s important contribution to home care of disabled elderly people, two limitations should be mentioned. The conduct of our study has been greatly affected due to the COVID-19. There were some difficulties in recruiting and contacting eligible participants. Therefore, the study had small samples, which limits the generalizability and representativeness. The sampling method may have resulted in selection and response bias. Family caregivers were recruited via purposive sampling through community nurses. Those participants were more likely to have a better relationship with community nurses. We communicated with community nurses in advance, in order to avoid having their present which could cause response bias. However, in a few cases, community nurses were present for a portion of the interview, and family caregivers could have felt more inclined to give a positive evaluation of interaction with community nurses.

## Conclusion

The interaction between family caregivers and community nurses will be very beneficial, which will reduce the burden of family caregivers and have positive impacts on the disabled elderly people through better care provision. These results inform recommendations for community nursing and home care for the disabled elderly people, and providing a new perspective that we can develop and implement intervention to facilitate positive interactions and bring the higher quality of care to older adults with disabilities.

## Data Availability

The datasets generated and/or analysed during the current study are not publicly available due to be the important data of the author's master's thesis, but are available from the corresponding author on reasonable request.
